# Ultrasound-Assisted Surface Modification of MWCNT Using Organic Acids

**DOI:** 10.3390/ma14010072

**Published:** 2020-12-25

**Authors:** Patricia A. de León-Martínez, Aidé Sáenz-Galindo, Carlos A. Ávila-Orta, Adalí O. Castañeda-Facio, Marlene L. Andrade-Guel, Uriel Sierra, German Alvarado-Tenorio, Juan Bernal-Martínez

**Affiliations:** 1 Departamento de Ciencia y Tecnología de Polímeros, Facultad de Ciencias Químicas, Universidad Autónoma de Coahuila, Ingeniero José Cárdenas Valdez S/N, República, Saltillo, Coahuila C.P. 25280, Mexico; p.leon@uadec.edu.mx (P.A.d.L.-M.); adali.castaneda@uadec.edu.mx (A.O.C.-F.); 2 Departamento de Materiales Avanzados, Centro de Investigación en Química Aplicada (CIQA), Blvd. Enrique Reyna Hermosillo, San José de los Cerritos, Apdo.140, Saltillo, Coahuila 25294, Mexico; marlene.andrade@ciqa.edu.mx (M.L.A.-G.); German.alvarado@ciqa.edu.mx (G.A.-T.); 3 Laboratorio Nacional de Materiales Grafénicos, Centro de Investigación en Química Aplicada (CIQA), Blvd. Enrique Reyna Hermosillo, San José de los Cerritos, Apdo.140, Saltillo, Coahuila 25294, Mexico; uriel.sierra@ciqa.edu.mx; 4 Unidad Médica Ojo Caliente, Cañada Honda 129, Ojo de Caliente 1, Aguascalientes C.P. 20196, Mexico; drjuanbernal@gmail.com

**Keywords:** MWCNT, surface modification, ultrasound, uremic toxins, organic acids

## Abstract

In the present work, multiple-wall carbon nanotubes (MWCNTs) were surface modified in an environmentally friendly way, using low-frequency ultrasonic energy. This type of modification was carried-out using two different types of organic acids, citric acid (CA) and oxalic acid (OA). The modification of the MWCNTs was confirmed by Fourier-transform infrared spectroscopy (FTIR), where functional groups such as OH, C=O, O–C=O and COOH were detected. By means of Raman spectroscopy, an increase in carbon surface defects was found. On the other hand, using X-ray photoelectron spectroscopy (XPS), oxidation was evidenced on the surface of the modified MWCNT. In both Raman spectroscopy and XPS, the results indicate a greater modification when CA is used, possibly due to the fact that CA has a larger number of functional groups. MWCNT-CA showed good dispersion in methanol, while MWCNT-OA showed good stability in methanol and ethanol. Finally, a 20% removal of creatinine efficiency improvement was found with respect to the unmodified MWCNTs, while no improvement was found in the case of urea and uric acid.

## 1. Introduction

Multiple-wall carbon nanotubes (MWCNTs) have been thoroughly studied and used as additives in polymeric materials, with the aim of enhancing properties such as electrical conduction, heat transport, and mechanical performance of base materials [1]. Furthermore, MWCNTs can act as adsorbents of residues or organic pollutants due to their chemical and structural nature [2,3,4,5]. For example, Xu et al. studied the removal of heavy metals in wastewater with surface modified MWCNT with nitric acid, finding that MWCNTs can potentially be used as adsorbents for this purpose [3]. On the other hand, studies in medical applications have also been carried out [6]. For example, Abidin et al. obtained a hemodialysis membrane based on MWCNT modified with citric acid and polyethersulfone (PES) and compared it with a commercial membrane. These authors found a 5% improvement in the removal of uremic toxins from bovine serum compared to commercial membranes [7]. In addition, Irfan et al. manufactured and evaluated membranes for hemodialysis based on PES and nanocomposites based on MWCNT modified with two acids (nitric and sulfuric acid) and polyvinylpyrrolidone (PVP). In this study, they compared membranes based on pure PES and PES with nanocomposite, finding that urea depuration increased by 59.2% and creatinine increased by 57.3%, compared to the membrane with pure PES. Therefore, these authors concluded that the mixture of PES and the nanocomposite improved the biocompatibility and the ability to remove uremic solutes [8].

As shown above, surface modification is necessary because MWCNTs tend to agglomerate due to their high cohesion forces, resulting in poor adhesion between the MWCNT and the polymer matrix, and therefore in in a poor dispersion. In turn, this results in the decrease of the final properties of the nanocomposite [9,10]. Besides, surface modification or functionalization has an important role in improving the dispersion stability and the interfacial interactions produced by chemical, physical or mechanical bonds between the MWCNT and the polymeric matrix [11,12,13,14]. On the other hand, ultrasound-assisted surface modification of MWCNT has been carried out using different media such as surfactants, oxidizing agents, using concentrated acids or organic acids, among others [14,15]. Various authors, such as Maleki and Mallakpour in 2017, have mentioned that ultrasound-assisted modification of MWCNT is a novel technique due to the advantages it presents, such as being an economic technique, with environmental benefits (green technology), selective oxidation, short reaction times, easy product isolation and easy preparation [16,17]. Surface modification using organic acids has attracted great interest, since the addition of chemical groups (OH, C=O, O–C=O and COOH) on the surface of MWCNT can be achieved. This type of modification favors better dispersion, processing and compatibility with other materials [14,18]. In 2018, Cabello et al. modified MWCNT using 1,4-diaminobutane and oxalic acid using ultrasonic homogenizer. The authors found an optimal degree of modification in MWCNT with 1,4-diaminobutane at a reaction time of 20 min and in oxalic acid at 32 min. Furthermore, the results showed structural damage in the MWCNT and resulted in a lower percentage of surface modification when reaction time is 64 min [19]. Sáenz et al. conducted surface modification of MWCNTs with three organic acids (maleic, malonic, and tartaric), performing the modification with ultrasonic energy, and found that the dispersion in polar solvents is maintained for more than 24 h. The authors found a greater modification with malonic acid, followed by modification with maleic acid and finally with tartaric acid modification [20]. Andrade et al. modified graphene nanoplates with a mixture of organic acid (citric acid-oxalic acid, citric acid-formic acid) with an ultrasonic homogenizer in continuous mode and plugged into a catenoidal titanium horn of 25 mm in diameter. Two different treatment times of 30 and 60 min were applied. All experiments were performed at room temperature, finding a removal of 75% of urea when the citric-oxalic acid mixture was used for 30 min and a removal of uric acid 35% in all the graphene samples modified with the citric-oxalic acid mixture [21].

Therefore, in recent studies, graphene and MWCNT have been successfully modified with acids and amines assisted with ultrasound and they have been tested for uremic toxins removal. The aim of this study is to chemically modify MWCNT surface with organic acids (citric acid and oxalic acid) with the use of a low-frequency ultrasound bath under mild conditions (40 °C for 180 min). Furthermore, the effect of adsorption on uremic toxins is studied as a possible application (urea, creatinine and uric acid).

## 2. Materials and Methods

### 2.1. Materials

Industrial grade MWCNTs were purchased from Cheaptubes, Cambridgeport, VT, USA, with a purity of 95%, an external diameter of 30–50 nm and a length of 5–20 microns were used. The organic acids used were citric acid with a purity of 99% (Sigma-Aldrich, St. Louis, MO, USA) and oxalic acid dihydrate with a purity of 99% (Fisher, Boston, MA, USA). Urea, creatinine, and uric acid with a purity of 99% (Sigma-Aldrich, St. Louis, MO, USA) were used.

### 2.2. Surface Modification of MWCNT

**Figure 1:**
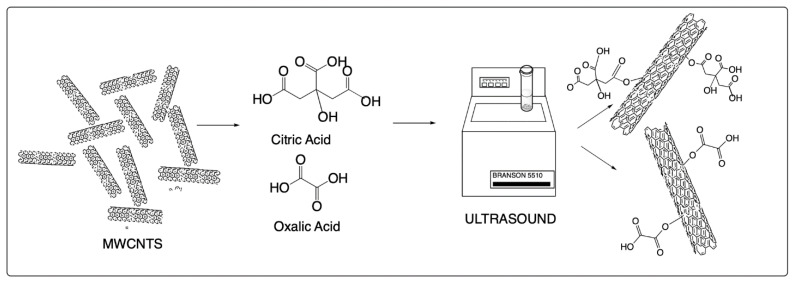
Ultrasound-assisted multiple-wall carbon nanotube (MWCNT) modification process with citric acid and oxalic acid.

A quantity of 200 mg of MWCNT were placed in test tubes, which were mixed in saturated aqueous solutions of each organic acid, citric acid, and oxalic acid. The amount of citric acid and oxalic acid in water varies due to the solubility of each acid, and since the solution used in this work is a saturated solution, the solubility will depend on the concentration and temperature. In the case of citric acid, a 1:1 ratio was used, while a 1:0.5 ratio was used for oxalic. After mixing, the MWCNTs with the saturated solutions of each of the acids, were placed in a ultrasound bath (BRANSON, Brookfield, CT, USA), at 40 kHz and 220 V, for 180 min at a temperature of 40 °C; after the sonication time, they were filtered and then vacuum dried for 24 h at 50 °C (see Figure 1).

### 2.3. Characterization Techniques

For FTIR a Thermo Nicolet MAGNA 550 (Thermo Fisher Scientific, Waltham, MA, USA) infrared spectrophotometer was used. The conditions under which these analyzes were carried-out were, 100 scans with a resolution of 16 cm^−1^, in the range of 400 to 4000 cm^−1^. Previously, the samples were dried in a vacuum oven (Perkin Elmer, San Diego, CA, USA) at 100 °C for 15 h, then KBr tablets were prepared and the samples analyzed. Raman spectroscopy was used to analyze the surface of MWCNTs using a Horiba Scientific Micro Raman Xplora spectrophotometer (Horiba Scientific, Kyoto, Japan) at 1000 to 4000 cm^−1^ and a laser at 532 nm with 50×. For XPS (X-ray photoelectron spectroscopy) an X-ray photoelectron spectroscopy Versa Probe II, (PHI, Chanhassen, MN, USA) was used. All spectra were collected using radiation (1486.6 eV). The alpha hemispherical analyzer was operated in the constant energy mode with survey scan pass energy of 117.4 eV. The high-resolution spectra were obtained using a pass energy of 11.75 eV. XPS was also used to assess the chemical bonding state and the elemental composition of the samples. The C1s peak (binding energies of C–O, C–N, etc.) was deconvoluted using Gaussian curves with no restriction of the position and area. To observe the surface of the MWCNTs, a TITAN JSM-7410 (FEI, Waltham, MA, USA) TEM (Transmission Electron Microscopy) was used, the samples were prepared by dispersion in acetone and sonication for 30 min. After this, a drop of the solution containing the dispersed nanostructures was placed on a carbon-coated copper grid (lacey carbon). Finally, the acetone was evaporated at room temperature. The dispersion tests were carried out at room temperature, 0.5 mg of the unmodified and modified MWCNT were weighted, placed in containers with 5 mL of different solvents, both polar and non-polar, such as water, methanol, ethanol, acetone, acetate, ethyl and hexane. The vials were sonicated for 5 min and left to rest for 24 h at room temperature. Uremic toxins adsorption. Urea, creatinine, and uric acid were dissolved in distilled water at different concentrations (20, 40, 60, 80, 100, 120, 140, 160 mg/L) to create a calibration curve, which was read on the spectrometer UV-Vis model UV-1800 (Shimadzu, Kyoto, Japan). Adsorption experiments were performed in beakers with 20 mL of 160 mg/L solution (urea, creatinine, or uric acid) and 50 mg of unmodified and modified MWCNT. The beakers were placed on a stir plate at 37 °C and with a stirring speed of 100 rpm for 4 h (typical hemodialysis treatment duration). Every 15 min a sample was taken off, and the absorbance was measured on the UV-Vis spectrophotometer. The experiments were carried-out twice and the data given are the average values. The concentration of uremic toxins in the solution was determined using the Beer-Lambert law by monitoring absorbance versus wavelength with a maximum of 200 and 293 nm for urea, creatinine, and uric acid.

The elimination percentage was calculated according to the following equation:

(1) $$\%Removal=\frac{\left(Ci-Cf\right)}{Ci}\times 100$$

where *Ci* is the initial concentration and *Cf* is the final concentration. The adsorption capacity of MWCNT was calculated with the following equilibrium equation:

(2) $$qe=\frac{\left(Ci-Cf\right)V}{m}\times 100$$

where *V* is volume in l L of solution and *m* is the adsorbent mass (mg).

## 3. Results and Discussion

**Figure 2:**
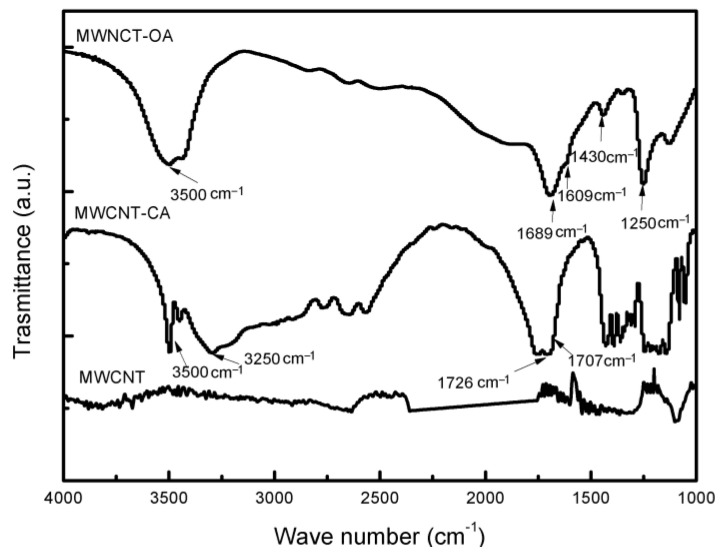
FTIR spectra of MWCNT, MWCNT-CA and MWCNT-OA.

Figure 2 shows the FTIR spectrum for MWCNT, in which no signal attributed to any functional group was presented. In the case of MWCNT-CA a signal in 3500 cm^−1^ is observed which is attributed to the stretching of the hydroxyl (OH) groups, in 3250 cm^−1^ to the stretching of the CH_2_ groups and in 1707 cm^−1^ the signal attributed to the stretching of the carbonyl groups (C=O). The signals between 1550 and 1300 cm^−1^ are attributed to the symmetrical stretching of the carboxyl groups (COOH), to the bending of the OH and to the movement of the CH groups. Finally, the signal at 1726 cm^−1^ is attributed to the stretching of the carbonyl groups of ester functional groups. The presence of ester bonds confirms the interaction between MWCNT and citric acid, according to what was reported by Abidin et al. [7]. The spectrum of MWCNT-OA show at 3500 cm^−1^ the signal attributed to the stretching of the OH groups, at 1430 cm^−1^ there is the symmetrical vibration of the C–O and C–C groups, in addition at 1250 cm^−1^, a symmetrical vibration of the bonds C–O and C=O is observed. Finally, two signals one at 1689 and the other at 1609 cm^−1^ were assigned to the vibrations of the C=O carbonyl groups [22]. As reported by Mendive et al. [23], two absorbance bands in said region refer to two types of carbonyl groups, for oxalic acid modification, shows evidence of a modification/oxidation on the surface of the MWCNT.

**Figure 3:**
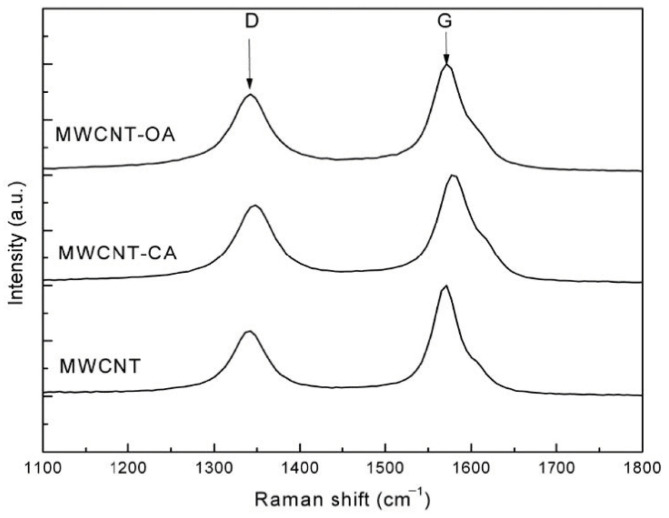
Raman spectra of MWCNT, MWCNT-CAMWCNT-CA and MWCNT-OA.

**Figure 4:**
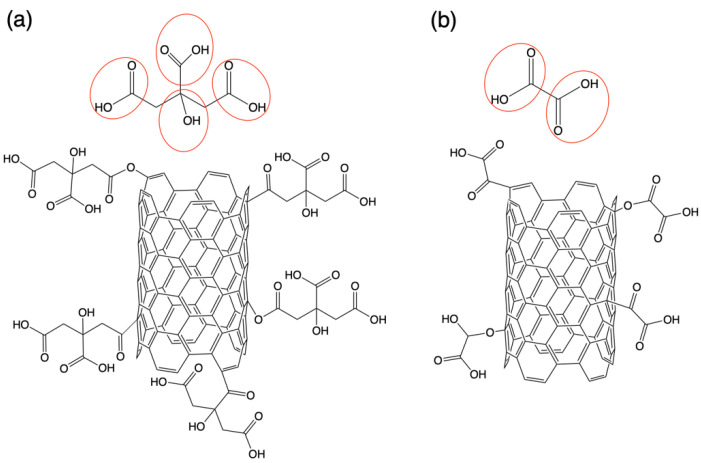
Possible chemical modification of MWCNT with (**a**) CA and (**b**) OA.

Figure 3 shows the Raman spectra of the MWCNT with and without modification. Two signals were observed in all cases corresponding to the D and G bands of nanotubes: D (1340 cm^−1^) and G (1580 cm^−1^) as well as three important effects, (i) a slight shift in the spectra of the modified MWCNTs from bands D and G towards higher wavelengths, which is indicative of a lower nanotube-nanotube interaction, according Bokobza et al. [24]. (ii) a change in signal width was also observed, which is attributed to a greater amount of amorphous carbon [25]. It is important to remark that both the displacement and the change in the signal width were greater in the case of MWCNT-CAMWCNT-CA, possibly due to the fact that citric acid has a greater number of functional groups which can be grafted to the structure of the MWCNT. Possible covalent bonds that can occur between the chemical groups of acids (-COOH, -COH and -OH) and MWCNT are shown in Figure 4, producing defective carbons in which the hybridization state changes from sp^2^ to sp^3^ bond [14,26]. (iii) The intensity ratio (I_D_/I_G_) was calculated to obtain the degree of order or defects present on the surface of the MWCNT. Table 1 shows the results of the ratio of I_D_/I_G_ signals for MWCNT (0.5882), MWCNT-CA (0.7284) and MWCNT-OA (0.7290), an increase in said ratio is indicative of surface defects carbon and low degree of graphitization [25,27,28]. Several authors, such as Jeon and Hamouma, suggest that the increase in the ratio of I_D_/I_G_ signals is a clear sign of covalent functionalization in MWCNTs with chemical groups [28,29].

Deconvolution of the C1s XPS spectra showed significant results since it possible to determine the type and proportion of functional groups present on the surface of the MWCNT. In Table 2, the carbon-oxygen ratio (C/O) is shown, for the unmodified MWCNT, having a ratio of 54.55, 2.62 for the MWCNT-CAMWCNT-CA and 8.49 for the MWCNT-OA. The decrease in C/O ratio is indicative of the oxidation of the MWCNTs, being mainly evident in the MWCNT-CAMWCNT-CA. Comparing the MWCNT with the MWCNT-CAMWCNT-CA and MWCNT-OA, a decrease in Csp^2^ was observed in both functionalized materials and only with the MWCNT-CAMWCNT-CA an increase in Csp^3^, while in the MWCNT-OA, the percentage of Csp^3^ was lower, possibly due to the presence of oxalic acid residue remaining in the material that was not bounded to the surface of the MWCNT.

**Figure 5:**
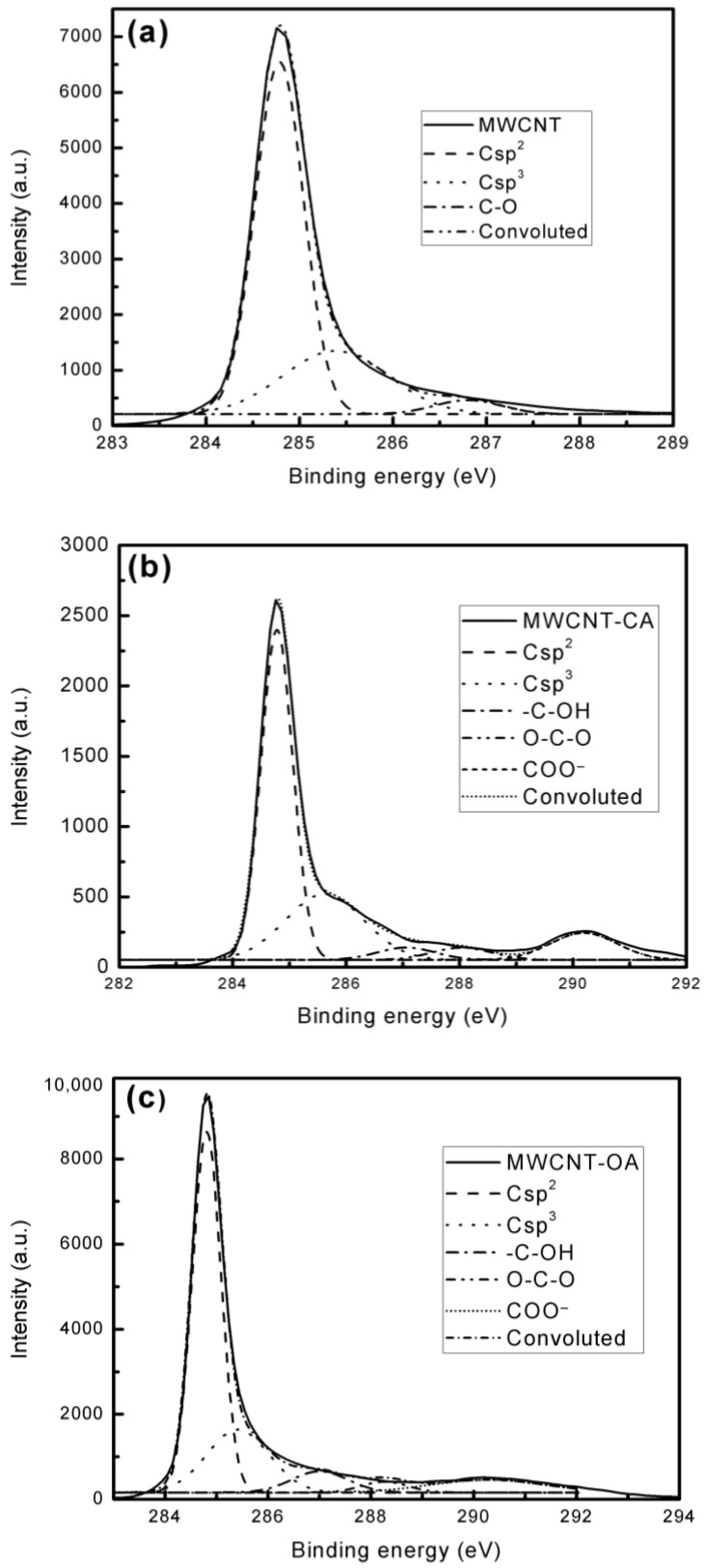
XPS C1s spectra and deconvoluted curves of: (**a**) MWCNT, (**b**) MWCNT-CA and (**c**) MWCNT-OA.

As can be seen in the XPS images (Figure 5a) the unmodified MWCNTs contains C–O (3.796%), while the MWCNT-CA (Figure 5b) contains functional groups such as C–O (3.084%), O–C–O (3.901%) and COO^-^ (9.570%), and in the case of MWCNT-OAMWCNT-OA the following C–O (6.852%), O–C–O (2.877%) and COO- (8.390%) groups were found. These results demonstrate that the method is suitable to achieve chemical modification of MWCNTs through the use of citric acid and oxalic acid; however, the results show that the oxidation and functionalization is greater in the case of citric acid compared to oxalic acid.

**Figure 6:**
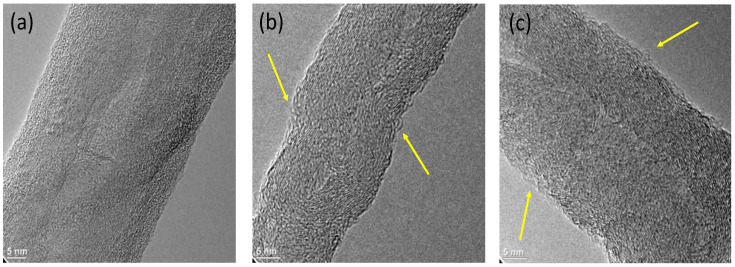
Transmission electron microscopy (TEM) images of (**a**) MWCNT, (**b**) MWCNT-CA and (**c**) MWCNT-OA.

Morphological characterization was carried-out using transmission electron microscopy (TEM), where images of MWCNT, MWCNT-CA and MWCNT-OA were obtained. In Figure 6a, the structure of an unmodified MWCNT is observed, which has a diameter of 42 nm and a smooth and homogeneous surface. On the other hand, in Figure 6b,c the images of the modified MWCNTs are shown, in which a rough surface is observed in the MWCNTs. Similar results were reported by different authors such as Kim et al. These authors reported that when modifying MWCNT with acids using ultrasound, the pure MWCNT show a smooth structure, while the modified MWCNT shows an evident roughness [30]. Price et al. argued that the oxidation of MWCNT with nitric and sulfuric acid assisted by ultrasound produces good dispersion of MWCNT in ethanol. The authors also mention in the study that, by increasing the time and intensity of the treatment, cavitation produces a deformation in the MWCNT and the high shear forces during sonication generate defects in the MWCNT [10]. Therefore, the surface chemical modification of MWCNTs with organic acids generated a rough surface during the treatment with ultrasound.

**Figure 7:**
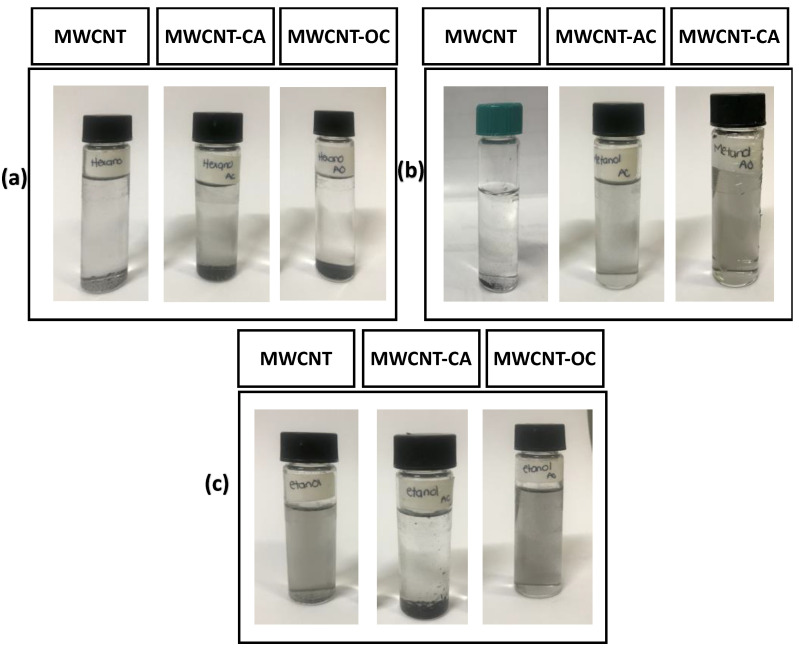
Dispersion of MWCNT, MWCNT-CA AND MWCNT-OA in different solvents (**a**) hexane, (**b**) methanol and (**c**) ethanol after 24 h.

Dispersion analysis at room temperature is useful to give evidence of modification of MWCNT quickly and easily. In both, MWCNTs with and without modification, a good dispersion was observed at time zero (t = 0) in all solvents after being sonicated for 5 min. However, after 24 h the unmodified MWCNT settled in all the solvents, this is due to the fact that no affinity is present (Figure 7a). On the other hand, the MWCNT-CA remain dispersed in methanol after 24 h (Figure 7b) and for the case of the MWCNT-OA, they showed good stability in both methanol and ethanol solvents (Figure 7b,c).

The dispersion in these solvents is possibly due to (i) functional groups provided hydrophilic properties on the surface of the MWCNTs [9,31,32]. (ii) presence of functional groups in the MWCNT reduces the Van der Waals interactions present between the MWCNTs, promoting the separation and dispersion of MWCNT in solvents such as ethanol as reported by Avilés [31]. (iii) when MWCNTs are loaded with chemical groups that present the same charge, it allows them to repel each other, keeping the solution dispersed and in colloidal stability in polar media, as reported by Lee and Farbod [33,34].

**Figure 8:**
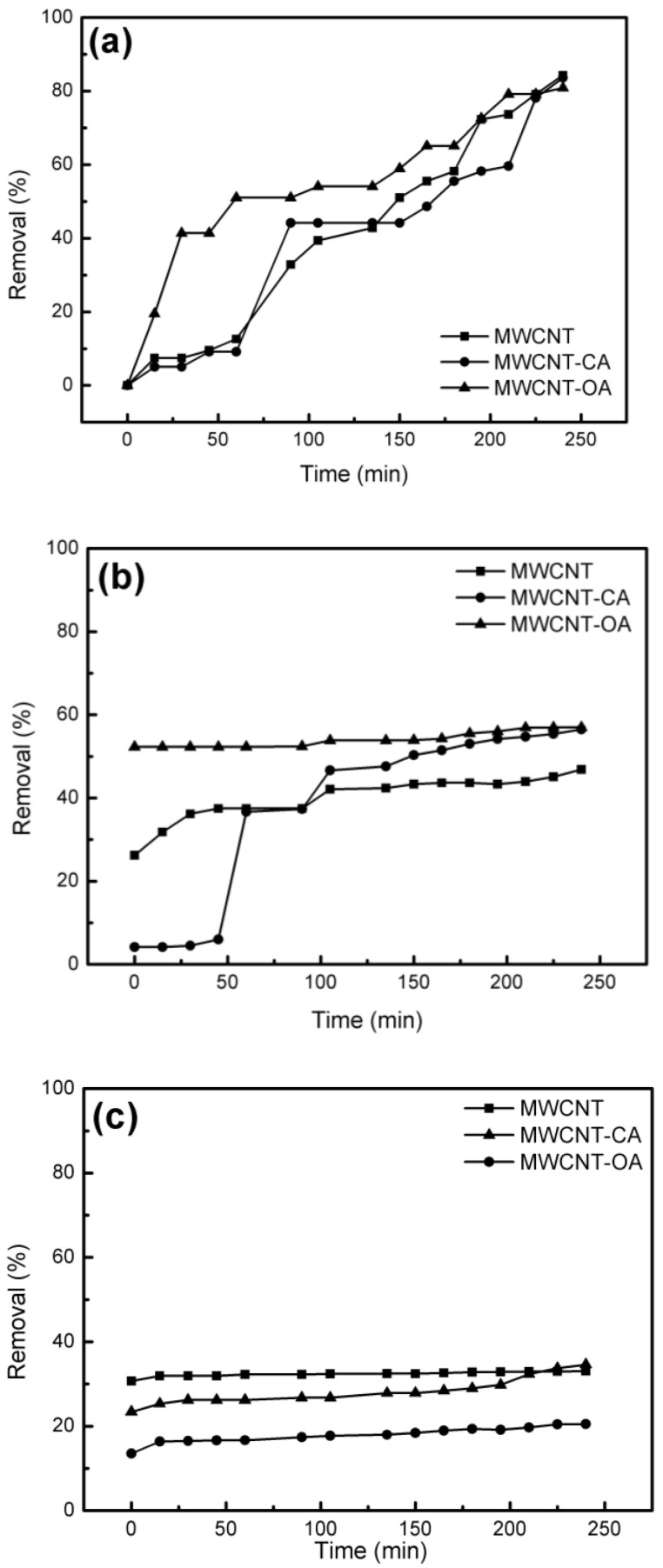
Removal percentage of (**a**) urea (**b**) creatinine and (**c**) uric acid with MWCNT, MWCNT-CA, MWCNT-OA.

**Figure 9:**
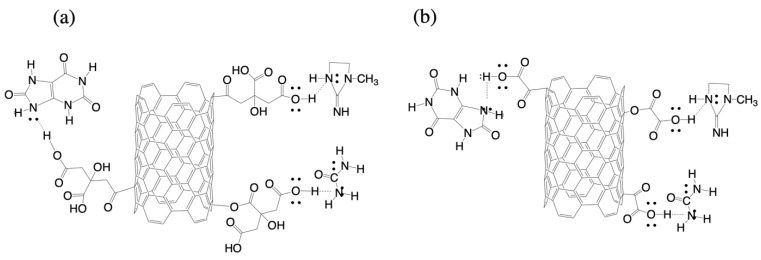
Possible interaction of (**a**) MWCNT-CA and (**b**) MWCNT-OA with uremic toxins.

Finally, the adsorption analysis of uremic toxins specifically urea, creatinine and uric acid was performed. Figure 8a shows the percentage removal of urea in solution for 4 h. In which, it is observed that in all cases there is a removal between 80–85% of toxins. However, in the case of modified MWCNTs the removal of urea with respect to time is slower than that of unmodified MWCNTs. The creatinine removal percentage of pure MWCNTs is 45%, and in the modified MWCNTs a 20% increase in creatinine removal was found (Figure 8b). In addition, removal over time is fast compared to the unmodified MWCNT. As reported by Eknoyan et al. in 2002, a hemodialysis membrane must remove at least 60% of urea and creatinine [35], so the values obtained are within range. On the other hand, the Figure 8c shows the percentage of uric acid removed by pure MWCNT which was 33%, while in the case of MWCNT-CA was 34.5% and 20.5% for MWCNT-OA. In the case of pure MWCNT and MWCNT-CA they have a similar behavior, however, pure MWCNT have a slight advantage in the elimination of uric acid with respect to time. Andrade et al., modified graphene with a mixture of organic acids (citric acid-oxalic acid and citric acid-formic acid), finding an elimination of citric acid-oxalic acid in a range of 30–50%, which attributes it to the structure of uric acid [21]. These authors suggested a mechanism in which by an attraction between the free electron pair of oxygen in the carbonyl functional group with the free electron pair of nitrogen of a urea molecule. At the same time, the hydroxyl functionality of the graphene surface could interact with the amine and carbonyl group of urea by means of bridging hydrogen bonds [6]. Later, Cabello et al. modified graphene nanoplates with amino groups and studied the removal of urea and uric acid. In their work they mention that the urea and uric acid molecules are adsorbed by electrostatic interactions, hydrogen bonding and π-π interactions [36]. Therefore, in this study, hydroxyl functionality of both citric acid and oxalic acid found on the surface of MWCNT could interact with the amine group of uremic toxins via hydrogen bridge-type linkages (Figure 9).

## 4. Conclusions

It can be concluded that the chemical modification was possible in an environmentally friendly way, using low-frequency ultrasonic energy. Sonochemical energy treatment with a saturated solution of organic acids led to an increase disorder or defects in the structure based on the MWCNTs. These defects were generated by introducing functional groups to the surface of the MWCNT such as OH, C=O, O–C=O and COOH as seen in the deconvoluted XPS signals. On the other hand, Raman and in XPS suggest a greater modification when citric acid is used, possibly due to the fact that citric acid has a larger number of functional groups. Additionally, it was also concluded that the surface modification of MWCNT can influence the adsorption behavior (time, % removal and selectivity) of MWCNT. MWCNTs show better performance than MWCNT-AC and MWCNT-AO for uric acid removal. However, MWCNT-AO shows better performance than MWCNT for creatinine adsorption, even at short treatment times. For MWCNT-AC, a better performance than MWCNT was found at long treatment times. For the removal of urea, at the end of the adsorption time, the adsorption is similar in all cases, however at short treatment times the MWCNT-AO show better performance and in the case of MWCNT-AC it presents better adsorption performance at long treatment times. It is important to reduce the uremic toxin removal time, since a potential application is in hemodialysis where an increase in efficiency is equal to a decrease in treatment time.

## Figures and Tables

**Table 1:** I_D_/I_G_ ratio of MWCNT, MWCNT-CA and MWCNT-OA obtained by Raman spectroscopy.

Sample	I_D_	I_G_	I_D_/I_G_
MWCNT	0.5859	0.9960	0.5882
MWCNT-CAMWCNT-CA	0.7286	1.0002	0.7284
MWCNT-OA	0.7261	0.9960	0.7290

**Table 2:** X-ray photoelectron spectroscopy (XPS) C1s deconvolution results of MWCNT, MWCNT-CA and MWCNT-OA.

Sample	C/O	Csp^2^ (%)	Csp^3^ (%)	C–O (%)	O–C–O (%)	COO^−^ (%)
MWCNT	54.55	69.136	27.068	3.796	-	-
MWCNT-CA	2.62	55.357	27.367	3.804	3.901	9.570
MWCNT-OA	8.49	59.412	22.469	6.852	2.877	8.390

## Data Availability

The data presented in this study are available on request from the corresponding author.
